# Selective ROCK Inhibitor Enhances Blood Flow Recovery after Hindlimb Ischemia

**DOI:** 10.3390/ijms241914410

**Published:** 2023-09-22

**Authors:** Hend Salah Fayed, Mouayad Zuheir Bakleh, Jasni Viralippurath Ashraf, Alison Howarth, Daniel Ebner, Ayman Al Haj Zen

**Affiliations:** 1 College of Health and Life Sciences, Hamad Bin Khalifa University, Doha P.O. Box 34110, Qatar; 2 Nuffield Department of Medicine, Target Discovery Institute, University of Oxford, Oxford OX3 7FZ, UK; 3 BHF Centre of Research Excellence, Division of Cardiovascular Medicine, Radcliffe Department of Medicine, University of Oxford, Oxford OX3 9DU, UK

**Keywords:** high content screening, kinase inhibitors, ROCK inhibitor, blood flow, limb ischemia

## Abstract

The impairment in microvascular network formation could delay the restoration of blood flow after acute limb ischemia. A high-content screen of a GSK-published kinase inhibitor library identified a set of ROCK inhibitor hits enhancing endothelial network formation. Subsequent kinase activity profiling against a panel of 224 protein kinases showed that two indazole-based ROCK inhibitor hits exhibited high selectivity for ROCK1 and ROCK2 isoforms compared to other ROCK inhibitors. One of the chemical entities, GSK429286, was selected for follow-up studies. We found that GSK429286 was ten times more potent in enhancing endothelial tube formation than Fasudil, a classic ROCK inhibitor. ROCK1 inhibition by RNAi phenocopied the angiogenic phenotype of the GSK429286 compound. Using an organotypic angiogenesis co-culture assay, we showed that GSK429286 formed a dense vascular network with thicker endothelial tubes. Next, mice received either vehicle or GSK429286 (10 mg/kg i.p.) for seven days after hindlimb ischemia induction. As assessed by laser speckle contrast imaging, GSK429286 potentiated blood flow recovery after ischemia induction. At the histological level, we found that GSK429286 significantly increased the size of new microvessels in the regenerating areas of ischemic muscles compared with vehicle-treated ones. Our findings reveal that selective ROCK inhibitors have in vitro pro-angiogenic properties and therapeutic potential to restore blood flow in limb ischemia.

## 1. Introduction

The formation of new blood vessels is critical in tissue repair and regeneration in response to injury such as ischemic insult. In response to reduced blood supply, the tissue attempts to compensate for it by triggering new blood vessel formation and remodeling (neovascularization) [1,2,3]. However, adaptive neovascularization mechanisms are often insufficient to restore blood flow fully, and the resulting tissue damage can lead to various ischemic diseases, including myocardial infarction, stroke, and peripheral artery disease [4,5,6,7]. Therefore, the stimulation of functional neovascularization has been proposed as a therapeutic strategy to treat vascular ischemic disease [8,9].

Many receptor kinases have been demonstrated to be essential in regulating new blood vessel formation. For instance, the vascular endothelial growth factor receptors (VEGFRs), platelet-derived growth factor receptors (PDGFRs), and fibroblast growth factor receptors (FGFRs) are key signaling pathways involved in many endothelial biological activities such as endothelial sprouting, proliferation, migration, and survival, all of which are essential for blood vessel formation [10,11,12]. Likewise, the angiopoietin/Tie2 pathway contributes to the endothelial remodeling and maturation of new blood vessels during angiogenesis [13,14]. The Eph receptor family, the largest family of tyrosine kinase receptors, play essential roles with their Ephrin ligands in vascular development and postnatal angiogenesis [15]. Moreover, the role of tyrosine kinase receptors in angiogenesis associated with disease has provided a solid rationale to target them for treating diseases such as cancer [16]. Multiple anti-angiogenic tyrosine kinase inhibitors such as sunitinib, sorafenib, and pazopanib have been approved by the US Food and Drug Administration (FDA) for targeted anti-cancer therapy [17,18]. In contrast, the clinical translation of pro-angiogenic growth factor therapies to treat vascular ischemic disease lags far behind anti-angiogenic therapies [19]. Many clinical trials using pro-angiogenic therapies, promoting neovascularization to restore blood flow in ischemic tissues, have focused on administrating vascular growth factors such as VEGFA [20], FGF2 [21], and HGF [22]. These growth factors were delivered either as recombinant proteins or as the cDNA encoding these proteins [23,24]. However, more success has yet to be achieved in clinical outcomes [25]. Possible reasons for this failure include the administered recombinant proteins or the overexpressed genes not reaching the optimum level of the growth factor required to be beneficial in the patients [26,27]. For instance, in VEGF clinical trials, an efficient pro-angiogenic response would be expected to be associated with tissue edema. However, this side effect of VEGF therapy was only observed in a few clinical studies [28,29].

An alternative approach is to use a small molecule that might be given systemically for a short period and could be repeated in cycles as necessary. Therefore, identifying new small molecules with pro-angiogenic properties might overcome the cost and conventional limitations of protein- or gene-based pro-angiogenic therapies [30]. To identify novel small molecules regulating angiogenesis, we screened the published protein kinase inhibitor set (PKIS) by GlaxoSmithKline (GSK) using an endothelial tube formation assay [31]. This screen identified 65 compounds that inhibit endothelial network formation and 10 compounds that enhance endothelial network formation. Six of the ten enhancer hits were ROCK inhibitor compounds. In this study, we have characterized these compounds and focused on investigating a selective ROCK inhibitor (GSK429286) as a candidate for enhancing endothelial network formation. We also evaluated the in vivo therapeutic potential of administering the selected ROCK inhibitor to improve tissue reperfusion in a mouse model of hindlimb ischemia.

## 2. Results

### 2.1. Phenotypic Drug Screen of Endothelial Tube Formation and Enhancer Hits Identification

**Figure 1:**
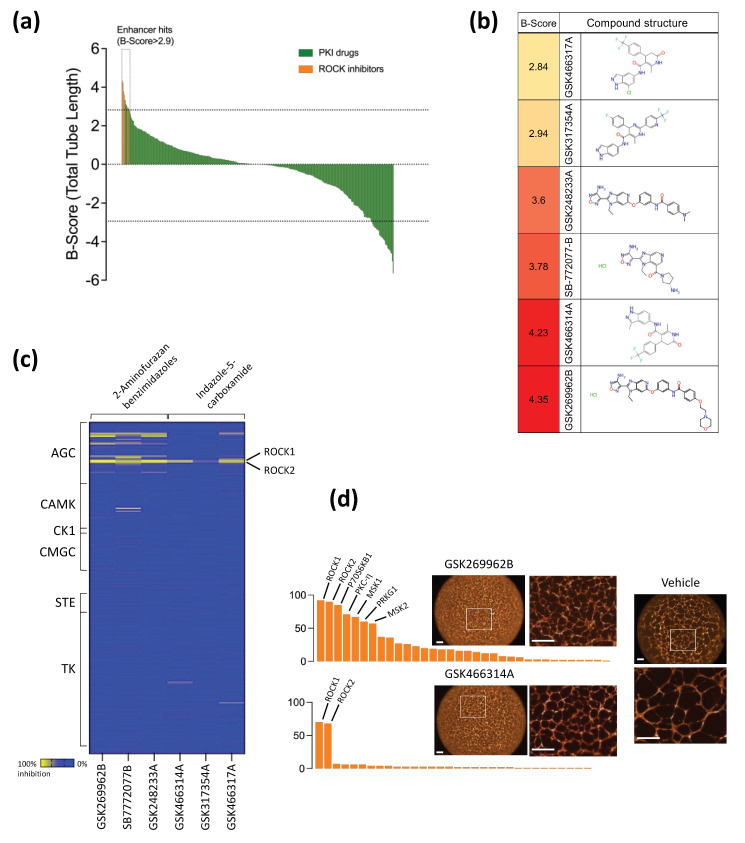
Phenotypic screen of GSK PKI compound library using endothelial tube formation and hit identification. (**a**) Bar chart showing the results of high-content screening. Total tube length was normalized to controls treated with vehicle (DMSO) using B-Score method. Enhancer hits of the total tube length are indicated above the dotted line (hit threshold was calculated as follows: median_(vehicle)_ + 5 × median absolute deviation_(vehicle)_). (**b**) Chemical structure of ROCK inhibitor compound hits. (**c**) Heatmap depicting the kinase inhibition activity of ROCK inhibitor hits against 224 protein kinases, assayed at 100 nM using Nanosyn assay. (**d**) Selectivity kinase inhibitor profiles of GSK269962B and GSK466314A at 100 nM identified in the phenotypic screening. Fluorescence images of endothelial tube formation produced by both compounds versus DMSO (vehicle). The white boxes showing the area where the high magnification images have been taken. Scale bar = 500 µm.

A phenotypic drug screen of the GSK PKI set was performed at a concentration of 10 μM to profile the kinase inhibitor effect on endothelial tube formation, as described in our previous study [31]. The screening was performed in duplicate with the overall Z′ value for all plates being more than 0.3. A compound was tagged as an enhancer hit if its B-Score was >2.9 [median_(vehicle)_ + 5 × median absolute deviation_(vehicle)_]. A total of ten compounds were identified as enhancer hits, of which six have Rho kinase (ROCK) as their primary target (Figure 1a). The ROCK inhibitor compound hits belonged to two chemotypes: aminofurazan and indazole (Figure 1b). Their kinase activity has been characterized through large panels of kinase inhibition using Nanosyn Caliper enzyme assay panels [31,32,33]. The panel represented 224 kinases (approximately 40% of the human protein kinome). The kinase inhibition activity of the six compounds (ROCK inhibitors) was evaluated at a concentration of 100 nM to evaluate their potency values and selectivity profiles (Figure 1c). indazoles were more selective to ROCK1 and ROCK2 than aminofurazans. While both chemotypes significantly enhanced the total tube length parameter of endothelial tube formation, the endothelial tubes treated with aminofurazan compounds showed a dense network with broadly flattened endothelial cells connected with only cellular protrusions, indicative of tube immaturity. In contrast, the endothelial tubes treated with indazoles showed more mature tubes composed of several elongated cells (Figure 1d). GSK466314A (indazole-based ROCK inhibitor) was prioritized based on its specificity and vascular-like network phenotype. Because the GSK466314A library compound was commercially unavailable, we have selected the GSK429286 compound synthesized by GSK with an identical chemical structure [34], which is commercially available for further validation and biological studies. Taken together, our phenotypic screen identified ROCK inhibitors as enhancers of endothelial tube formation.

### 2.2. GSK429286 Promotes Endothelial Tube Formation

**Figure 2:**
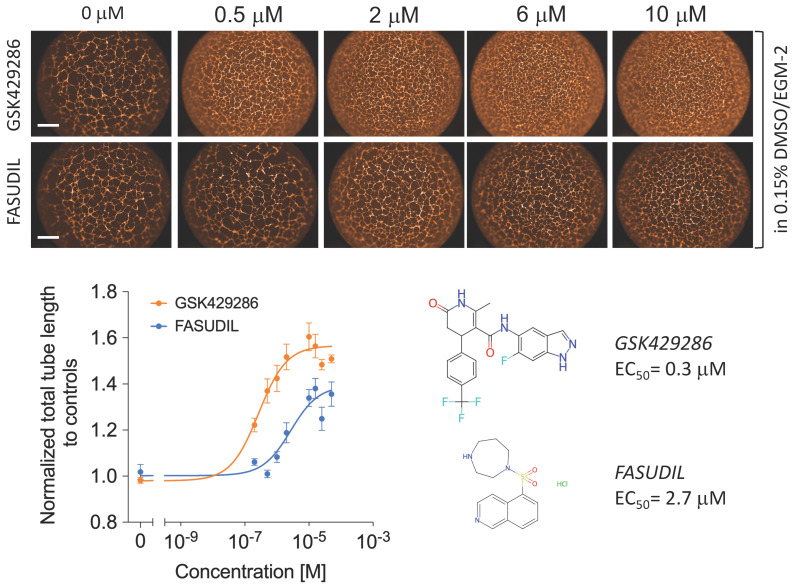
Dose–response curve for total tube length demonstrating the effect of GSK429286 and Fasudil on endothelial tube formation assay. **Upper**, representative fluorescent images of endothelial tubes stained with phalloidin (actin, orange). Total tube length was normalized to controls. The solid lines are best fits to the experimental data using a logistic function in Prism GraphPad software. Data are expressed as mean ± SD. n = 4 per concentration. Scale bar = 500 μm.

**Figure 3:**
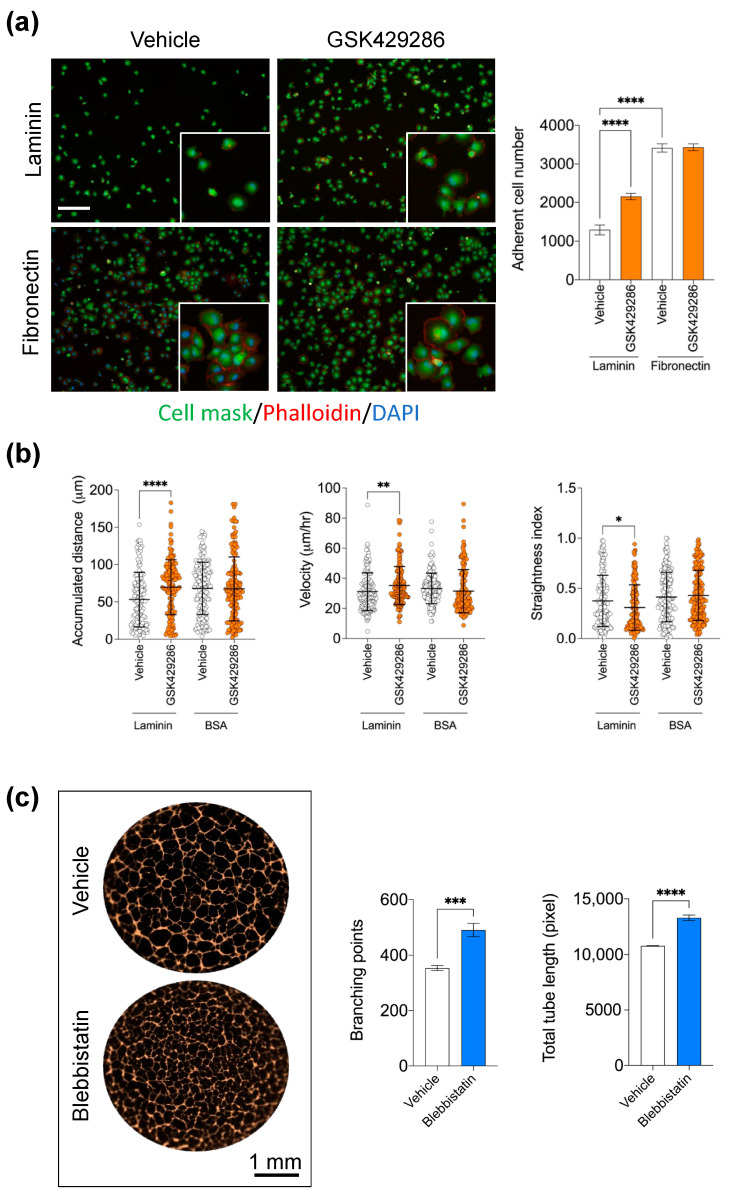
Effects of the ROCK inhibitor (GSK429286) on endothelial cell adhesion and motility. (**a**) Effect of GSK429286 (10 μM) on Laminin- and fibronectin-mediated HUVEC adhesion. **Left**, representative images confirming the cell spreading after 3 h of seeding. Data are expressed as mean ± SD; **** *p* < 0.0001 compared to Vehicle (DMSO) (n = 6 replicates, one-way ANOVA followed by Bonferroni post hoc test). Scale bar = 100 μm. (**b**) Effect of GSK429286 on endothelial cell motility. Graphs show the accumulated distance, cell velocity, and the straightness index for HUVEC cultured in the presence of GSK429286 (10 μM) or vehicle (DMSO) on laminin or cultured on BSA. Cells were imaged with objective 20× and their positions tracked for 3 h with 5 min intervals using live high-content imaging (Operetta). Data are expressed as mean ± SD; Mann–Whitney test, * *p* < 0.05, ** *p* < 0.01, and **** *p* < 0.0001 compared to Vehicle. n = 150–180 cells per condition. (**c**) Effect of blebbistatin, a myosin II inhibitor, on endothelial tube formation in HUVEC. **Left**, representative images of endothelial tubes treated with blebbistatin (5 μM) and vehicle (DMSO) and stained with phalloidin (orange). Scale bar = 1 mm. **Right**, Quantification of total tube length and branching points parameters for each condition. Data are expressed as mean ± SD; *** *p* < 0.001 and **** *p* < 0.0001 compared to Vehicle (DMSO) (n = 6 replicates, unpaired *t* test).

**Figure 4:**
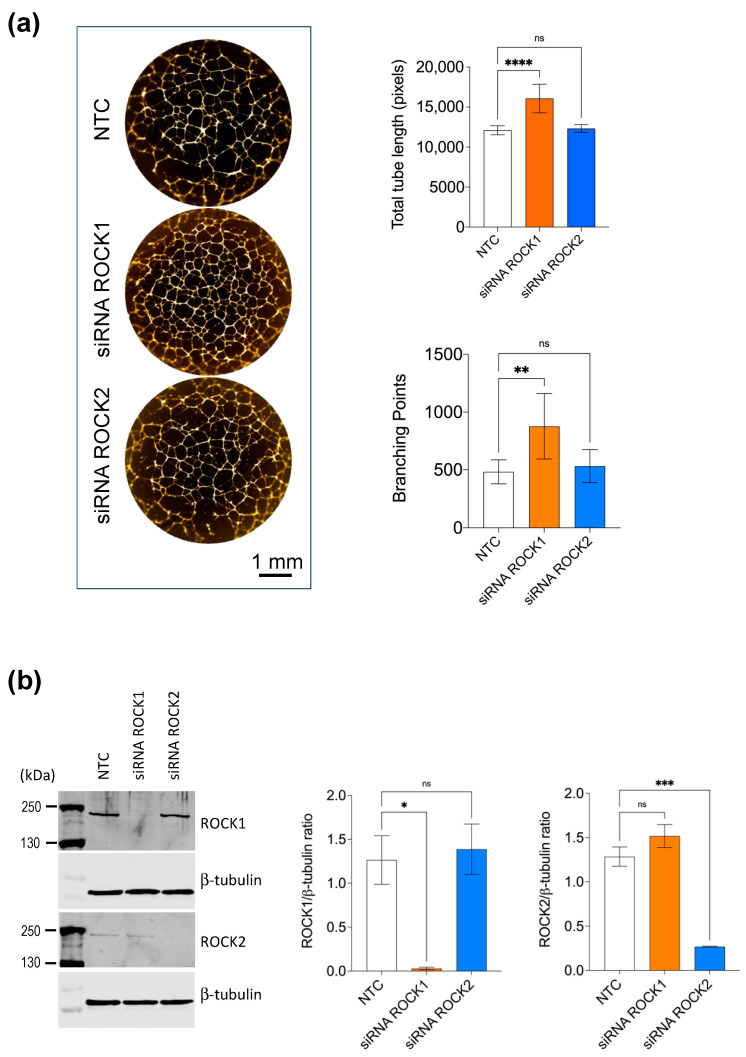
Effect of ROCK1 and ROCK2 isoform knockdown on endothelial tube formation. (**a**) HUVEC were transfected with Non-Targeting Control (NTC) siRNA, ROCK1 siRNA, or ROCK2 siRNA and subjected to Matrigel tube formation assay. **Left**, representative fluorescent images of endothelial tubes stained with phalloidin (actin, orange). Scale bar = 1 mm. **Right**, Quantification of total tube length and branching points parameters for each condition. Data are represented as arbitrary units corresponding to pixels detected and are expressed as mean ± SD; n = 6 per condition; one-way ANOVA followed by Bonferroni post hoc test, ** *p* < 0.01 and **** *p* < 0.0001 compared to NTC. (**b**) Representative Western blots of lysates from transfected HUVECs to validate the knockdown efficiency with NTC, siRNA ROCK1, and siRNA ROCK2 at 72 h after transfection. **Right**, Western blots were quantified by densitometry using Image J version 1.54e NIH Software. The protein expression of ROCK1 and ROCK2 was normalized with respect to the corresponding β-tubulin signals. Data are expressed as mean ± SD; ns is abbreviation for non-significant; * *p* < 0.05; *** *p* < 0.001 compared to NTC (n = 3 independent biological replicates, one-way ANOVA followed by Bonferroni post hoc test).

The primary screening was performed at a single compound concentration (10 μM). Therefore, next, we validated the efficacy of the GSK429286 compound at nine concentrations, decreasing from 15 μM to generate a dose–response curve. In this experiment, the dose–response effect of the compound was also compared to a classic ROCK inhibitor (Fasudil) with a different chemotype to assess the drug’s potency and efficacy. We found that GSK429286 was more potent and efficient compared to Fasudil, with an average EC_50_ of 0.3 μM (Figure 2). A time course experiment of tube formation revealed that the enhancing effect of the ROCK inhibitor on endothelial branching was detected when it was incubated for the first three hours or longer after cell seeding. However, when the incubation of the ROCK inhibitor was started after 3 h of seeding, no significant effect of ROCK inhibition could be detected anymore (Figure S1). Cell–matrix adhesion and cell migration are two key cellular processes in the early stage of endothelial tube formation [35,36]. The interaction between extracellular matrix (ECM) and endothelial cells is important in modulating endothelial branching and patterning during endothelial tube formation [37,38]. To test whether ROCK inhibition affects cell adhesion, HUVECs were treated with GSK429286 or vehicle and then seeded in wells with pre-coated laminin (the main extracellular matrix component of the Matrigel) or fibronectin. Fibronectin served as a control since Matrigel does not contain fibronectin. The compound significantly enhanced HUVEC cell adhesion to laminin, but we did not detect any difference in cell adhesion to fibronectin between GSK429286-treated cells and controls (Figure 3a). To examine the effects of the GSK429286 inhibitor on cell migration, time-lapse live cell tracking was performed on GSK429286-treated cells and vehicle-treated cells seeded on laminin or BSA. We found that GSK429286 significantly increased the endothelial cell velocity and the accumulated distance compared to controls. In contrast, the endothelial cell displacement analysis revealed that ROCK inhibitor-treated cells showed a reduction in the mean straightness index compared to controls, indicating that they were less directional than controls (Figure 3b). ROCK regulates the cell motility and directional migration by inducing contractility through the phosphorylation of myosin II [39]. To determine whether the inhibition of myosin II impacts the endothelial tube network like GSK429286, we examined the pharmacological inhibition of myosin II by Blebbistatin on tube formation. Blebbistatin enhances the tube formation producing the phenotype of branched networks like those of the ROCK inhibitor (Figure 3c). The compound GSK429286 inhibits both ROCK isoforms: ROCK 1 and 2. Despite ROCK 1 and ROCK2 sharing more than 90% structural similarity, several previous reports have suggested that they have distinct roles and different regulation [40,41]. To determine whether the inhibition of ROCK1 or ROCK2 enhances endothelial tube formation, the effect of ROCK1 or ROCK2 inhibition on tube formation was evaluated separately, using small interfering RNA (siRNA). ROCK1 knockdown, but not ROCK2 knockdown, significantly enhanced both parameters of endothelial tube formation: total tube length and branching (Figure 4a). The efficiency and specificity of the siRNA silencing were assessed by the protein abundance of two kinase isoforms (Figure 4b). Western blotting showed that the expression levels of ROCK1 and ROCK2 are inhibited in siRNA ROCK1- and ROCK2-treated cells, respectively. Importantly, ROCK2 expression levels were not affected by siRNA ROCK1 and the ROCK2 expression levels were not affected by siRNA ROCK1, indicating the specificity of siRNA knockdown.

**Figure 5:**
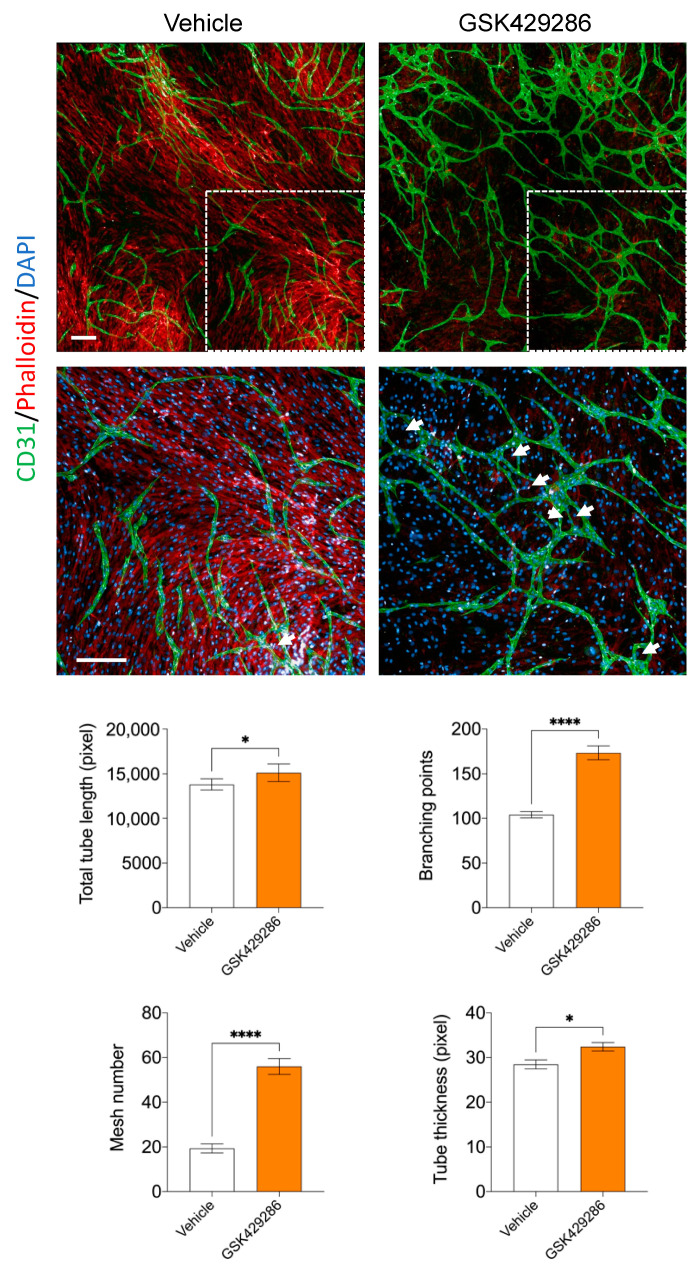
Effect of GSK429286 on angiogenesis using an organotypic co-culture assay. HUVECs are co-cultured with human aortic smooth muscle cells at final concentration of 5 µM. On day 7, cocultures were fixed and stained with an antibody against CD31 (endothelial cells), phalloidin (actin), and DAPI (nuclei). White arrows: mesh network. The dotted white boxes showing the area where the high magnification images below have been taken. Nine fields were quantified for each well (n = 6 wells per group). Error bars, mean ± SD, unpaired *t* test, * *p* < 0.05; **** *p* < 0.0001 compared with vehicle. Scale bar = 200 μm.

While the endothelial tube formation assay captures many aspects of in vivo angiogenesis, such as cellular adhesion, cell migration, tubulogenesis, and branching morphogenesis, it does not capture other important aspects of angiogenesis, such as cell proliferation, vessel stability, or interaction with other cell types [42]. Therefore, we used a secondary angiogenesis assay to validate the angiogenic phenotype of GSK429286 in a more complex cellular setting, where endothelial cells were co-cultured with vascular smooth muscle cells for seven days [43]. GSK429286 promotes the formation of endothelial tubes with a significant appearance of meshes compared to controls. In contrast to the Matrigel tube formation assay, the tubes were significantly thicker than the controls (Figure 5).

### 2.3. GSK429286 Compound Enhanced Blood Flow Recovery after Ischemia

**Figure 6:**
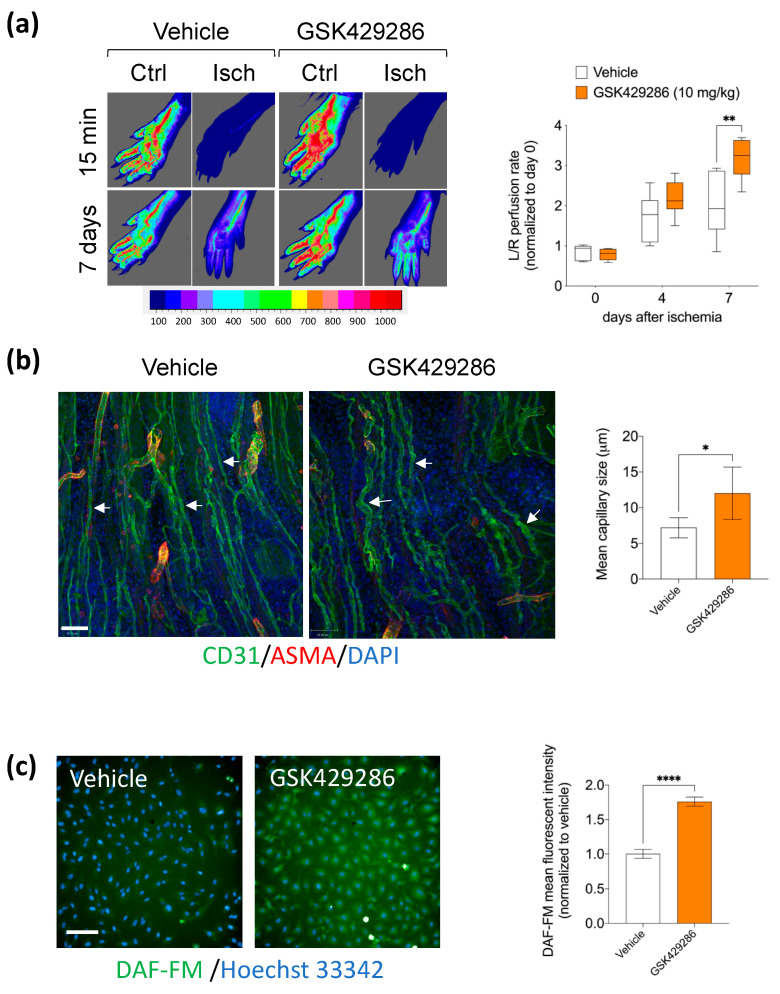
Effect of GSK429286 administration on blood flow recovery after limb ischemia. Experimental design: mice were subjected to limb ischemia (day 0) at which time mice were randomized and injected i.p. daily with either vehicle or GSK429286 for one week. (**a**) Representative images of blood flow measured by a Laser Speckle Contrast Imager (dark blue = low blood flow, red = high blood flow, min = minutes). **Right**, quantitative analysis of blood flow in the paws (n = 6 per group). Data were normalized to day 0 value for each animal. Data are presented as box plots, with maximum, minimum, and quartile range. Two-way ANOVA (** *p* < 0.01) followed by Sidak’s multiple comparisons test, ** *p* < 0.01 compared with Vehicle group. (**b**) Representative confocal microscopy images of thick longitudinal sections for the ischemic adductor muscles from mice treated with GSK429286 or vehicle at day 7 after ischemia. Microvessels (CD31, green), smooth muscle cells (ASMA, red), and nuclei (DAPI, blue) are shown. Effect of GSK429286 administration on the new capillary (arrows) size quantified in the regenerating zone of ischemic adductor muscles. n = 6 muscles per group; three fields per muscle were studied. Unpaired *t* test, * *p* < 0.05 compared with vehicle group. Scale bar = 56 μm. (**c**) HUVEC were incubated with GSK429286 (10 μM) or vehicle (DMSO) overnight. Next, HUEVC were loaded with DAF-2 DA for 30 min to visualize nitric oxide production. Next, intracellular fluorescence signal was measured in nine fields for each well (n = 6 wells per group). Error bars, mean ± SD, unpaired *t* test, **** *p* < 0.0001 compared with vehicle. Scale bar = 50 μm.

To address the physiological relevance of the ROCK inhibitor on adult vascular morphogenesis and blood flow, we tested the effect of GSK249286 administration in the mouse model of hindlimb ischemia. Mice were injected intraperitoneally with GSK429286 of a 10 mg/kg dose daily for one week after ischemia induction. Next, we measured the impact of the GSK429286 compound on the blood flow, and found that the recovery of paw perfusion was improved in mice treated with GSK429286 compared to controls. This effect reached statistical significance on day seven after surgery (Figure 6a). Next, we assessed the 3D structure of new microvessels in regenerating areas of ischemic muscles on day seven and found that elevated blood flow in mice treated with the compound was associated with an increased capillary diameter in the ischemic muscles (Figure 6b). Several studies showed that Rho GTPase, via its downstream effector ROCK, downregulated eNOS gene expression and activity [44,45]. Since nitric oxide (NO) is a potent vasodilator and contributes to the blood flow recovery following limb ischemia [46,47], we evaluated the effect of the compound GSK429286 on NO production by endothelial cells. As expected, GSK429286 significantly increased intracellular NO production in cultured HUVECs compared with the Vehicle (Figure 6c). Thus, the GSK429286 compound improved blood flow recovery after ischemia during treatment. At the same time, it showed increased capillary size in the regenerating muscle areas.

## 3. Discussion

The morphology of the new blood vessel network determines its efficiency for transporting sufficient nutrients and oxygen to the damaged tissue after ischemia [48]. The endothelial tube formation assay is widely used as a phenotypic assay to study the effects of various compounds or growth factors on vascular network morphogenesis [49,50,51]. In the current study, we identified hit compounds that enhance the formation of a highly branched endothelial network, some with structural similarity and some which are primary targets to known ROCK inhibitors. Two distinct chemical structures of ROCK inhibitor hit compounds were identified: aminofurazan-based inhibitors [52] and indazole-based inhibitors. Based on the kinase selectivity profiling, two compounds showed high selectivity against ROCK1 and ROCK2 isoforms belonging to indazole-based inhibitors compared to other hit compounds. The indazole derivatives have been used as specific kinase inhibitors, including tyrosine kinase and serine/threonine kinases [53]. A novel class of indazole-based small molecules identified to target ROCK were highly potent and selective ROCK inhibitors. These developed compound series have also shown improved oral bioavailability, and significantly reduced mean arterial pressure in spontaneously hypertensive rats [34]. In our study, the indazole-based ROCK inhibitor (GSK429286) showed significantly enhanced potency, increasing endothelial network formation in vitro compared with the widely studied ROCK inhibitor Fasudil.

Our findings are consistent with a previous study using ROCK inhibitors, such as Y-27632 or Fasudil, indicating that ROCK inhibitors promote in vitro endothelial branching morphogenesis [54]. The possible mechanism responsible for ROCK inhibitor-mediated branching increase is partially due to its effects of actomyosin contraction, actin cytoskeleton reorganization [55], and cell–matrix adhesion [56]. Our data suggest that the inhibition of the activity of myosin II, a primary substrate of ROCK, triggers the formation of a dense endothelial network mimicking a similar phenotype of ROCK inhibition. Myosin II is dynamically localized to the endothelial cortex, and its increasing contracting activity inhibits the extension of filopodia protrusions. A previous study reports that the local downregulation of myosin II-mediated cortical contraction initiates pseudopodium in endothelial cells that mediate branching morphogenesis [57,58]. We have additionally shown that GSK429286 enhances endothelial cell adhesion on laminin, the main extracellular macromolecule of the Matrigel. The enhancing effect of the ROCK inhibitor on cell–matrix adhesion could contribute to stabilizing the endothelial protrusions induced by myosin II inhibition during endothelial tube formation [59]. This process would be important to link the actin cytoskeleton to the surrounding extracellular matrix, influencing the directional migration and thus modulating branching. Indeed, in our study, we found that the inhibition of ROCK activity led to increased endothelial cell motility, but the cells showed less directional migration than control cells. Several reports showed that endothelial cells treated with ROCK inhibitors failed to close the wound gap compared to controls [60]. In a wound healing migration assay, directional cell migration is essential to close the wound gap effectively. ROCK inhibitors impair cell polarization, leading to random cellular protrusions and motility [55]. Therefore, the randomness of cell migration with a high motility level might increase the incidence of ROCK-treated endothelial cells connecting with other neighboring cells, leading to an increase in the anastomosis formation rate. Using an RNAi-mediated suppression approach, we found that the loss of ROCK1, but not ROCK2, is sufficient to promote a similar angiogenic phenotype to that of the ROCK inhibitor compound. This detected effect is not due to the difference in the cellular expression level since we and others confirmed that cultured human endothelial cells express both isoforms [61]. Although the two isoforms share more than 90% sequence homology in their kinase domain, their regulatory domains at the C terminus demonstrate important divergence [62,63], and ROCK1 and ROCK2 knockout mice showed different phenotypes [64,65]. Shi J et al. demonstrated that ROCK isoforms have distinct roles in regulating the actin cytoskeleton [40]. Indeed, their findings support that ROCK1 is involved in destabilizing the actin cytoskeleton through regulating myosin II phosphorylation and peripheral actomyosin contraction, whereas ROCK2 is required for stabilizing the actin cytoskeleton through regulating cofilin phosphorylation. Therefore, the enhancing effect of the GSK429286 ROCK inhibitor on endothelial branching morphogenesis could be mediated by ROCK1 isoform inhibition contributing to the downregulation of myosin II phosphorylation. However, further experiments are required to confirm the specificity of this mechanism.

Accumulating investigations have indicated that the inhibition of ROCK signaling has potential beneficial effects on cardiovascular disorders such as coronary and cerebral vasospasm [66,67], hypertension [68] and pulmonary hypertension [69], and stroke [70]. Fasudil has been approved for specific clinical use in Japan to treat cerebral vasospasm after a subarachnoid hemorrhage [71]. Nevertheless, Fasudil and its metabolite “hydroxyfasudil” exhibit non-specific targeting of other serine/threonine kinases such as protein kinase A (PKA) and PKC [72]. In this study, we demonstrated that the systemic administration of ROCK inhibitor “GSK429286” improved blood flow recovery after limb ischemia, which could be partially attributed to the increase in the size of new microvessels in the regenerated muscles. We also detected a similar increase in endothelial tube thickness when endothelial cells were co-cultured with vascular smooth muscle cells. It has been shown that the loss of ROCK signaling in the endothelium results in the overexpansion of the blood vessel lumen in mouse embryos via modulating the activity of myosin II [73]. Previous studies have reported that capillary pericytes can regulate capillary diameter [74], and the exposure to ischemia leads to pericyte contraction [75]. It has been shown that ROCK inhibition reduces the contraction of capillary pericytes and increases capillary diameter [76]. The increase in microvessel size in the regenerating muscle areas could also be the consequence of the ROCK inhibitor effect on the pre-capillary smooth muscle cells. ROCK can directly regulate vascular smooth muscle cell contraction through myosin II phosphorylation, independently of the Ca^2+^/calmodulin-activated myosin light chain kinase pathway [77]. Indeed, previous animal studies reported that the pharmacological inhibition of myosin II causes a significant relaxation of the pre-capillary smooth muscle cells, contributing to the improvement in cerebral blood reperfusion after ischemic stroke [78]. Also, ROCK inhibition contributes to smooth muscle cell phenotype switching from a contractile into a synthetic phenotype [79]. Phenotypic switching of the contractile smooth muscle cells to a synthetic state is a critical cellular event that sustains the growth and outward remodeling of collaterals after ischemia [80].

Blood flow increase could be linked to the functional vasodilator property of ROCK inhibitors. In vivo pharmacological ROCK inhibition showed anti-inflammatory and vasodilatory activity [81]. In this study, we confirmed that the ROCK inhibitor “GSK429286” induced the production of nitric oxide (NO), a potent vasodilator, by cultured endothelial cells. These results are consistent with a previous study’s findings showing that ex vivo aortic rings from ROCK1^+/–^ and ROCK2^+/–^ mice produced higher levels of NO under basal conditions [82]. In contrast, ROCK and its upstream effector protein, small GTPase RhoA, decrease eNOS expression and NO production [45]. Of note, NO is a critical endothelium-derived relaxing factor for vascular networks after ischemia injury [83].

Protein kinases have been important targets for drug discovery, with more than 25 small molecules approved by the FDA and hundreds more in clinical testing phases to treat many diseases, including cancer, autoimmune disorders, and inflammatory conditions [84]. In this study, we identified a selective indazole-based ROCK inhibitor, GSK429286, as a candidate to enhance blood flow recovery after acute limb ischemia. While mouse hindlimb ischemia is valuable as a pre-clinical model of peripheral arterial disease (PAD), mimicking many specific features of PAD [85], we acknowledge the limitation of the acute induction nature of the model since PAD in patients is a chronic process which develops because of a gradual build-up of atherosclerosis. Thus, further in vivo studies should be set up to investigate the potential therapeutic effects of indazole-based ROCK inhibitors, and their mechanism of action, on the outcome of chronically induced ischemia in older animal cohorts with comorbidities to facilitate clinical translation [86] and to further define the underlying mechanisms and relevant pharmacological parameters to accelerate therapeutic translation.

## 4. Materials and Methods

### 4.1. High Content Screening of PKI GSK Library

The phenotypic drug screen analyzed in the current study was conducted using an image-based screen of a 367-compound PKI library (GSK) described previously [31,51]. The GSK Published Kinase Inhibitor Set (PKIS) is a collection of 367 kinase inhibitors previously published by GSK scientists [87]. Briefly, primary human umbilical vein endothelial cells (HUVECs, pooled donors) were purchased from Lonza and maintained in endothelial cell growth medium-2 (EGM-2, Lonza, Morristown, NJ, USA). First, Matrigel (354248, BD Bioscience, Haryana, India) was added into the 96-well plates and then incubated at 37 °C, 5% CO_2_ for 45 min. After Matrigel polymerization, PKIS library compounds were added on Matrigel with a final concentration of 10 μM. Next, a resuspension of HUVEC, passage 4, was prepared using Accutase (Sigma, Kanagawa, Japan). HUVECs were then seeded on the Matrigel with added compounds at a density of 15,000 cells per well. Compounds, media, and cells were dispensed using an automated liquid handling robot (JANUS, Denver, CO, USA) and cell dispenser (FlexDrop PLUS). The screen plates were incubated for ten hours at 37 °C, 5% CO_2_. The screen was performed in duplicate under a complete vascular growth culture medium (EGM-2). The screen plates were fixed with 4% PFA/PBS solution and stained with Alexa Fluor 568 phalloidin (Invitrogen, Carlsbad, CA, USA) to visualize the endothelial tubes. The plates were imaged with a 2× objective using a high-content imaging system (Operetta, PerkinElmer, Waltham, MA, USA). The 16-bit images were processed, segmented, and quantified using fully automated Metamorph image analysis software version 7.7.7.0 (Molecular Devices, Silicon Valley, CA, USA) [51]. Vehicle (DMSO) and Suramin (inhibitor of endothelial tube formation) were used in the original primary screen as controls, and added simultaneously with the PKI library compounds in each screen plate. The controls were used to calculate Z′ factor to assess the screen performance [88]. B-score was used as a method to normalize the total tube length parameter. The normalization was performed in the CellHTS2 web-based platform [89]. The B-score normalization method reduces the data variability plate-to-plate by calculating the residual values from the median polish and the median absolute deviation of the screen data.

### 4.2. siRNA Transfection

ROCK1 and ROCK2 knockdown in HUVECs was performed using pre-designed ON-TARGETplus™ smart-pool siRNAs from Dharmacon. ON-TARGETplus non-targeting siRNAs (NTC) were used as a negative control. Briefly, 120,000 cells/well were reverse transfected in 6-well plate format at final siRNA concentrations of 24 nmol/L pre-incubated with 6 µL Lipofectamine RNAiMAX (ThermoFisher Scientific, Waltham, MA, USA) in Opti-MEM media. Cell culture media were changed 24 h after transfection to avoid transfection reagent toxicity. Cells were used for further cell assays or protein analysis three days after transfection.

### 4.3. Endothelial Tube Formation Assay

An endothelial tube formation assay was performed as previously described [42]. Briefly, Matrigel (50 μL) was added to each well of a 96-well plate. Compounds and vehicle (DMSO) were first added to the polymerized Matrigel, and HUVECs with a density of 15,000 cells per well were then added to the top of the Matrigel. In siRNA experiments, transfected HUVECs were resuspended and added with a density of 15,000 cells per well on top of polymerized Matrigel. After incubation for eight to ten hours, endothelial tubes were fixed and stained with Alexa 568-conjugated Phalloidin (ThermoFisher Scientific). Next, endothelial tubes were imaged at 2× magnification using a high-content imaging (Operetta, Paris, France) system. The total tube length and branching points were assessed using Metamorph image analysis software.

### 4.4. Western Blotting

Cell lysis was obtained from siRNA-transfected cells after 72 h using a mixture of RIPA buffer (Sigma, St. Louis, MO, USA) and protease/phosphatase cocktail inhibitor (Roche, Indianapolis, IN, USA). Protein concentration was measured with a BCA assay (Pierce, Bradenton, FL, USA). Equal amounts of protein (5 μg) per sample were separated via SDS-PAGE and transferred to an Immobilon-P PVDF membrane (Millipore, Burlington, MA, USA). Membranes were blocked with 5% skimmed milk. The membranes were incubated with primary antibodies, monoclonal mouse anti-human β-tubulin (Sigma; 1:1000), monoclonal rabbit anti-human ROCK1 (Cell Signaling, Danvers, MA, USA; 1:1000), and monoclonal rabbit anti-human ROCK2 (Cell Signaling; 1:1000), followed by incubation with secondary antibodies, goat anti-mouse IRDye 800CW and goat anti-rabbit IRDye 680CW (1:5000, LiCOR, Lincoln, NE, USA), at room temperature for 3 h using iBind Western Device (ThermoFisher Scientific). Next, the membranes were imaged using an Odyssey infrared imaging system (LI-COR Biosciences, Lincoln, NE, USA). A densitometry analysis of protein expression was performed using the Image J gel analysis tool.

### 4.5. Endothelial Cell Adhesion Assay

A cell suspension of HUVEC was prepared at a density of 10,000 cells per well in basal endothelial cell culture media (EBM, Lonza) with no serum and growth factors. Compound GSK429286 (10 μM) or DMSO (Vehicle) were added to the cell suspension. HUVECs were seeded onto 10 μg/mL of laminin- or fibronectin-coated 96-well plates, where the adherent cells were captured. Cells were incubated for 15 min at 37 °C and 5% CO_2_. Next, the wells were gently washed five times with PBS to remove unbound cells using a multi-channel pipettor [90,91]. Fresh basal media with no serum/growth factors were added to the adherent cells, and were incubated for an additional three hours at 37 °C and 5% CO_2_. The plates were fixed with 4% PFA and stained with Alexa 568 conjugated phalloidin, deep red cell mask, and DAPI. Next, the plates were imaged and quantified using a high-content fluorescence imaging system (Operetta, Paris, France). The adherent cell number was quantified in nine identically positioned fields of each well using Harmony image analysis software version 3.5 (Perkin Elmer, Waltham, MA, USA).

### 4.6. Endothelial Cell Motility Assay

HUVECs were seeded with a density of 5000 cells per well in 10 μg/mL laminin- or BSA-coated 96-well plates. Cells were grown in EGM-2 media overnight. Next, CellTracker™ Orange CMTMR Dye (1 μM) was incubated with HUVEC for 30 min. After washing with PBS, HUVECs were pre-treated with GSK429286 (10 μM) or DMSO for one hour. To assess endothelial cell motility, time-lapse live imaging was conducted using 20× objective in a humidified, heated, and CO_2_-controlled and integrated chamber within the high-content imaging system (Operetta). The time-lapse imaging was run at 5 min intervals for a total period of 3 h. Two fields per well were imaged. At least 50 individual cells per well were analyzed, and the cell position was tracked automatically over time within the field image using the built-in Harmony image analysis software. Three parameters were calculated to evaluate the migratory cell behavior [92,93] using the same software: (1) the mean accumulated distance of the migrating cells over the experiment period; (2) the mean velocity of migrating cells; and (3) the straightness index was calculated by the ratio of the net displacement of a cell to the total migration length (accumulated distance). This measurement examined the straightness of cell trajectories (directionality).

### 4.7. Co-Culture Organotypic Angiogenesis Assay

Adult human aortic smooth muscle cells (Thermo Fisher Scientific Inc.) were seeded at a density of 20,000 cells per well. The next day, HUVECs were plated at density of 5000 cells per well on the confluent smooth muscle monolayer in a 96-well plate as previously described [51]. GSK429486 (5 μM) or DMSO as a control was added one day after the plating of HUVECs. The co-cultures were sustained under full growth media (EGM-2) for seven days. Endothelial tubes were visualized via immunofluorescence using a mouse anti-human CD31 monoclonal antibody (R&D systems) at 1:400 dilution and goat Alexa 488-conjugated anti-mouse IgG secondary antibody (Thermo Fisher Scientific Inc.) at 1:500 dilution. Actin fibers were stained with Alexa 568-conjugated Phalloidin (Thermo Fisher Scientific Inc.). Nuclei were visualized via DAPI staining. The endothelial tubes were imaged automatically at 10× magnification using the high-content imaging system (Operetta). Nine identically positioned fields were acquired from each well in each experiment. The following quantification of endothelial network features, total tube length, branching points and mesh number, was performed automatically using an NIH Image J-based tool (angiogenesis analyzer) [94]. Meanwhile, the tube thickness feature was quantified automatically using Harmony image analysis software (Perkin Elmer).

### 4.8. Hindlimb Ischemia Model and Blood Flow Measurement

All animal procedures were carried out according to the UK Home Office regulations and under appropriate project and personnel licenses. Briefly, 12-week-old female CD-1 strain mice were anesthetized with isoflurane. An incision was made in the skin overlying the middle portion of the left hind limb. The common femoral artery was exposed and dissected from the vein and nerve in the distal direction. The femoral artery was ligated proximally and distally of the intervening segment, as previously described [48,95]. The ligated segment was then excised by electro-coagulation. Animals were randomly divided into two groups. Immediately after femoral ligation, the animals were injected intraperitoneally with 10 mg/kg GSK429286 in 300 μL Saline/2.5% DMSO solution or vehicle (2.5% DMSO) (n = 6 per group). The compound dose was selected based on previous studies [34,96]. Paw perfusion was monitored using a Laser Speckle Contrast Imager (LSCI, Moor Instruments, Axminster, UK) as previously reported [97]. The FLPI measurements were made in a warm (24 °C) and quiet environment. The CCD camera was positioned 30 cm above the limb. The contrast images were processed to produce a color-coded live flux image (red denoted high perfusion, blue signified low perfusion) using the moorFLPI-2 measurement module (Moor Instruments). Measurements were made in the paw area of non-ligated and ligated sides before and immediately after limb ischemia. Follow-up measurements were acquired at 4 and 7 days after ischemia.

### 4.9. Whole-Mount Immunohistochemistry of Ischemic Muscles

Mice were euthanized seven days after ischemia induction. Adductor muscles were fixed under pressure, carefully dissected under a stereomicroscope, and snap frozen. Frozen muscle sections of 150 μm were prepared and stained as previously described [98]. The samples were stained with primary antibody rat anti-mouse CD31 (Pharmagen, Lahore, Pakistan; 1:100) overnight at 4 °C followed by incubation with the appropriate secondary antibody, Cy3-conjugated α-smooth muscle (SM) actin (Sigma; 1:100), and DAPI (ThermoFisher Scientific Inc.). Whole-mount muscle imaging was acquired on an LSM 710 Zeiss confocal microscope. Maximum-projection confocal images of the adductor muscle microvasculature were generated from z stacks acquired starting at the medial surface of the adductor muscle specimens. The new capillaries were defined as the microvessels that were not surrounded by smooth muscle cells (α-SM actin negative vessels) found in the regenerative regions of ischemic muscle fibers. The quantification of microvessel width was measured from three fields per muscle sample using Image J software (NIH).

### 4.10. Nitric Oxide Detection Assay

HUVECs were pre-incubated with GSK429286 (10 μM) in a 96-well plate overnight. Next, cell permeable fluorescent Nitric Oxide (NO) indicator, 4-amino-5-methylamino-2′, 7′-difluorescein diacetate “DAF-FM-DA” (Thermo Fisher Scientific Inc.) was added at a final concentration of 2 µM for 30 minutes, as previously reported [99,100]. Next, cells were washed, and NO production was visualized at excitation 460/490 and emission 500/550 using a high content fluorescence imaging system (Operetta). Intracellular fluorescence intensity was quantified in nine fields of each well using Harmony image analysis software (Perkin Elmer). Mean intracellular fluorescence was calculated by subtracting the integrated fluorescence density value obtained for the cell and the mean fluorescence of background readings.

### 4.11. Statistical Analysis

All statistical tests, graphic plots, and heatmaps were performed using GraphPad Prism software (version 9.0.2). One-way analysis of variance (ANOVA, New Providence, NJ, USA) was conducted for comparisons between more than two experimental groups. Two-way ANOVA was performed for comparisons with two experimental variables. Both analyses were followed by Bonferroni’s or Sidak’s multiple comparisons test analyses based on the method recommended by Prism 9. A non-parametric Mann–Whitney analysis was carried out for the cell motility comparison tests since the data were not normally distributed. In the other experiments, after passing the Kolmogorov–Smirnov normality test, an unpaired *t*-test was used to compare two group means with the two-tailed option. Data are expressed as mean ± standard deviation (SD) unless otherwise specified. The number of biological replicates used in each experiment is stated in the figure legends. *p* value < 0.05 was considered statistically significant.

## Data Availability

Not applicable.

## Supplementary Materials

The following supporting information can be downloaded at https://www.mdpi.com/article/10.3390/ijms241914410/s1.

## References

[1] Potente M., Gerhardt H., Carmeliet P. (2011). Basic and therapeutic aspects of angiogenesis. Cell.

[2] Ware J.A., Simons M. (1997). Angiogenesis in ischemic heart disease. Nat. Med..

[3] Semenza G.L. (2010). Vascular responses to hypoxia and ischemia. Arterioscler. Thromb. Vasc. Biol..

[4] Gjedde A., Kuwabara H., Hakim A.M. (1990). Reduction of functional capillary density in human brain after stroke. J. Cereb. Blood Flow Metab..

[5] White S.H., McDermott M.M., Sufit R.L., Kosmac K., Bugg A.W., Gonzalez-Freire M., Ferrucci L., Tian L., Zhao L., Gao Y. (2016). Walking performance is positively correlated to calf muscle fiber size in peripheral artery disease subjects, but fibers show aberrant mitophagy: An observational study. J. Transl. Med..

[6] Sano M., Minamino T., Toko H., Miyauchi H., Orimo M., Qin Y., Akazawa H., Tateno K., Kayama Y., Harada M. (2007). p53-induced inhibition of Hif-1 causes cardiac dysfunction during pressure overload. Nature.

[7] Duscha B.D., Kraus W.E., Jones W.S., Robbins J.L., Piner L.W., Huffman K.M., Allen J.D., Annex B.H. (2020). Skeletal muscle capillary density is related to anaerobic threshold and claudication in peripheral artery disease. Vasc. Med..

[8] Banfi A., von Degenfeld G., Gianni-Barrera R., Reginato S., Merchant M.J., McDonald D.M., Blau H.M. (2012). Therapeutic angiogenesis due to balanced single-vector delivery of VEGF and PDGF-BB. FASEB J..

[9] Annex B.H., Cooke J.P. (2021). New Directions in Therapeutic Angiogenesis and Arteriogenesis in Peripheral Arterial Disease. Circ. Res..

[10] Ferrara N., Gerber H.P., LeCouter J. (2003). The biology of VEGF and its receptors. Nat. Med..

[11] Adams R.H., Alitalo K. (2007). Molecular regulation of angiogenesis and lymphangiogenesis. Nat. Rev. Mol. Cell Biol..

[12] Jeltsch M., Leppanen V.M., Saharinen P., Alitalo K. (2013). Receptor tyrosine kinase-mediated angiogenesis. Cold Spring Harb. Perspect. Biol..

[13] Augustin H.G., Koh G.Y., Thurston G., Alitalo K. (2009). Control of vascular morphogenesis and homeostasis through the angiopoietin-Tie system. Nat. Rev. Mol. Cell Biol..

[14] Nasarre P., Thomas M., Kruse K., Helfrich I., Wolter V., Deppermann C., Schadendorf D., Thurston G., Fiedler U., Augustin H.G. (2009). Host-derived angiopoietin-2 affects early stages of tumor development and vessel maturation but is dispensable for later stages of tumor growth. Cancer Res..

[15] Adams R.H., Wilkinson G.A., Weiss C., Diella F., Gale N.W., Deutsch U., Risau W., Klein R. (1999). Roles of ephrinB ligands and EphB receptors in cardiovascular development: Demarcation of arterial/venous domains, vascular morphogenesis, and sprouting angiogenesis. Genes Dev..

[16] Claesson-Welsh L., Welsh M. (2013). VEGFA and tumour angiogenesis. J. Intern. Med..

[17] Gotink K.J., Verheul H.M. (2010). Anti-angiogenic tyrosine kinase inhibitors: What is their mechanism of action?. Angiogenesis.

[18] Qin S., Li A., Yi M., Yu S., Zhang M., Wu K. (2019). Recent advances on anti-angiogenesis receptor tyrosine kinase inhibitors in cancer therapy. J. Hematol. Oncol..

[19] Rust R., Gantner C., Schwab M.E. (2019). Pro- and antiangiogenic therapies: Current status and clinical implications. FASEB J..

[20] Isner J.M., Pieczek A., Schainfeld R., Blair R., Haley L., Asahara T., Rosenfield K., Razvi S., Walsh K., Symes J.F. (1996). Clinical evidence of angiogenesis after arterial gene transfer of phVEGF165 in patient with ischaemic limb. Lancet.

[21] Lederman R.J., Mendelsohn F.O., Anderson R.D., Saucedo J.F., Tenaglia A.N., Hermiller J.B., Hillegass W.B., Rocha-Singh K., Moon T.E., Whitehouse M.J. (2002). Therapeutic angiogenesis with recombinant fibroblast growth factor-2 for intermittent claudication (the TRAFFIC study): A randomised trial. Lancet.

[22] Morishita R., Makino H., Aoki M., Hashiya N., Yamasaki K., Azuma J., Taniyama Y., Sawa Y., Kaneda Y., Ogihara T. (2011). Phase I/IIa clinical trial of therapeutic angiogenesis using hepatocyte growth factor gene transfer to treat critical limb ischemia. Arterioscler. Thromb. Vasc. Biol..

[23] Deev R., Plaksa I., Bozo I., Mzhavanadze N., Suchkov I., Chervyakov Y., Staroverov I., Kalinin R., Isaev A. (2018). Results of 5-year follow-up study in patients with peripheral artery disease treated with PL-VEGF165 for intermittent claudication. Ther. Adv. Cardiovasc. Dis..

[24] Deev R.V., Bozo I.Y., Mzhavanadze N.D., Voronov D.A., Gavrilenko A.V., Chervyakov Y.V., Staroverov I.N., Kalinin R.E., Shvalb P.G., Isaev A.A. (2015). pCMV-vegf165 Intramuscular Gene Transfer is an Effective Method of Treatment for Patients with Chronic Lower Limb Ischemia. J. Cardiovasc. Pharmacol. Ther..

[25] Beltran-Camacho L., Rojas-Torres M., Duran-Ruiz M.C. (2021). Current Status of Angiogenic Cell Therapy and Related Strategies Applied in Critical Limb Ischemia. Int. J. Mol. Sci..

[26] Yla-Herttuala S., Markkanen J.E., Rissanen T.T. (2004). Gene therapy for ischemic cardiovascular diseases: Some lessons learned from the first clinical trials. Trends Cardiovasc. Med..

[27] Tongers J., Roncalli J.G., Losordo D.W. (2008). Therapeutic angiogenesis for critical limb ischemia: Microvascular therapies coming of age. Circulation.

[28] Makinen K., Manninen H., Hedman M., Matsi P., Mussalo H., Alhava E., Yla-Herttuala S. (2002). Increased vascularity detected by digital subtraction angiography after VEGF gene transfer to human lower limb artery: A randomized, placebo-controlled, double-blinded phase II study. Mol. Ther..

[29] Rajagopalan S., Mohler E.R., Lederman R.J., Mendelsohn F.O., Saucedo J.F., Goldman C.K., Blebea J., Macko J., Kessler P.D., Rasmussen H.S. (2003). Regional angiogenesis with vascular endothelial growth factor in peripheral arterial disease: A phase II randomized, double-blind, controlled study of adenoviral delivery of vascular endothelial growth factor 121 in patients with disabling intermittent claudication. Circulation.

[30] Simons M., Ware J.A. (2003). Therapeutic angiogenesis in cardiovascular disease. Nat. Rev. Drug Discov..

[31] Elkins J.M., Fedele V., Szklarz M., Abdul Azeez K.R., Salah E., Mikolajczyk J., Romanov S., Sepetov N., Huang X.P., Roth B.L. (2016). Comprehensive characterization of the Published Kinase Inhibitor Set. Nat. Biotechnol..

[32] Perrin D., Fremaux C., Shutes A. (2010). Capillary microfluidic electrophoretic mobility shift assays: Application to enzymatic assays in drug discovery. Expert. Opin. Drug Discov..

[33] Rudolf A.F., Skovgaard T., Knapp S., Jensen L.J., Berthelsen J. (2014). A comparison of protein kinases inhibitor screening methods using both enzymatic activity and binding affinity determination. PLoS ONE.

[34] Goodman K.B., Cui H., Dowdell S.E., Gaitanopoulos D.E., Ivy R.L., Sehon C.A., Stavenger R.A., Wang G.Z., Viet A.Q., Xu W. (2007). Development of dihydropyridone indazole amides as selective Rho-kinase inhibitors. J. Med. Chem..

[35] Lamalice L., Le Boeuf F., Huot J. (2007). Endothelial cell migration during angiogenesis. Circ. Res..

[36] Zanivan S., Maione F., Hein M.Y., Hernandez-Fernaud J.R., Ostasiewicz P., Giraudo E., Mann M. (2013). SILAC-based proteomics of human primary endothelial cell morphogenesis unveils tumor angiogenic markers. Mol. Cell. Proteom..

[37] Mongiat M., Andreuzzi E., Tarticchio G., Paulitti A. (2016). Extracellular Matrix, a Hard Player in Angiogenesis. Int. J. Mol. Sci..

[38] Dixelius J., Jakobsson L., Genersch E., Bohman S., Ekblom P., Claesson-Welsh L. (2004). Laminin-1 promotes angiogenesis in synergy with fibroblast growth factor by distinct regulation of the gene and protein expression profile in endothelial cells. J. Biol. Chem..

[39] Barbier L., Saez P.J., Attia R., Lennon-Dumenil A.M., Lavi I., Piel M., Vargas P. (2019). Myosin II Activity Is Selectively Needed for Migration in Highly Confined Microenvironments in Mature Dendritic Cells. Front. Immunol..

[40] Shi J., Wu X., Surma M., Vemula S., Zhang L., Yang Y., Kapur R., Wei L. (2013). Distinct roles for ROCK1 and ROCK2 in the regulation of cell detachment. Cell Death Dis..

[41] Hartmann S., Ridley A.J., Lutz S. (2015). The Function of Rho-Associated Kinases ROCK1 and ROCK2 in the Pathogenesis of Cardiovascular Disease. Front. Pharmacol..

[42] Al Haj Zen A. (2019). In Vitro Models to Study the Regulatory Roles of Retinoids in Angiogenesis. Methods Mol. Biol..

[43] Evensen L., Micklem D.R., Link W., Lorens J.B. (2010). A novel imaging-based high-throughput screening approach to anti-angiogenic drug discovery. Cytometry A.

[44] Laufs U., Liao J.K. (1998). Post-transcriptional regulation of endothelial nitric oxide synthase mRNA stability by Rho GTPase. J. Biol. Chem..

[45] Ming X.F., Viswambharan H., Barandier C., Ruffieux J., Kaibuchi K., Rusconi S., Yang Z. (2002). Rho GTPase/Rho kinase negatively regulates endothelial nitric oxide synthase phosphorylation through the inhibition of protein kinase B/Akt in human endothelial cells. Mol. Cell Biol..

[46] Bode-Boger S.M., Boger R.H., Alfke H., Heinzel D., Tsikas D., Creutzig A., Alexander K., Frolich J.C. (1996). L-arginine induces nitric oxide-dependent vasodilation in patients with critical limb ischemia. A randomized, controlled study. Circulation.

[47] Yu J., deMuinck E.D., Zhuang Z., Drinane M., Kauser K., Rubanyi G.M., Qian H.S., Murata T., Escalante B., Sessa W.C. (2005). Endothelial nitric oxide synthase is critical for ischemic remodeling, mural cell recruitment, and blood flow reserve. Proc. Natl. Acad. Sci. USA.

[48] Al Haj Zen A., Oikawa A., Bazan-Peregrino M., Meloni M., Emanueli C., Madeddu P. (2010). Inhibition of delta-like-4-mediated signaling impairs reparative angiogenesis after ischemia. Circ. Res..

[49] Arnaoutova I., George J., Kleinman H.K., Benton G. (2009). The endothelial cell tube formation assay on basement membrane turns 20: State of the science and the art. Angiogenesis.

[50] Arnaoutova I., Kleinman H.K. (2010). In vitro angiogenesis: Endothelial cell tube formation on gelled basement membrane extract. Nat. Protoc..

[51] Al Haj Zen A., Nawrot D.A., Howarth A., Caporali A., Ebner D., Vernet A., Schneider J.E., Bhattacharya S. (2016). The Retinoid Agonist Tazarotene Promotes Angiogenesis and Wound Healing. Mol. Ther..

[52] Stavenger R.A., Cui H., Dowdell S.E., Franz R.G., Gaitanopoulos D.E., Goodman K.B., Hilfiker M.A., Ivy R.L., Leber J.D., Marino J.P. (2007). Discovery of aminofurazan-azabenzimidazoles as inhibitors of Rho-kinase with high kinase selectivity and antihypertensive activity. J. Med. Chem..

[53] Tandon N., Luxami V., Kant D., Tandon R., Paul K. (2021). Current progress, challenges and future prospects of indazoles as protein kinase inhibitors for the treatment of cancer. RSC Adv..

[54] Kroll J., Epting D., Kern K., Dietz C.T., Feng Y., Hammes H.P., Wieland T., Augustin H.G. (2009). Inhibition of Rho-dependent kinases ROCK I/II activates VEGF-driven retinal neovascularization and sprouting angiogenesis. Am. J. Physiol. Heart Circ. Physiol..

[55] Amano M., Nakayama M., Kaibuchi K. (2010). Rho-kinase/ROCK: A key regulator of the cytoskeleton and cell polarity. Cytoskeleton.

[56] Pipparelli A., Arsenijevic Y., Thuret G., Gain P., Nicolas M., Majo F. (2013). ROCK inhibitor enhances adhesion and wound healing of human corneal endothelial cells. PLoS ONE.

[57] Fischer R.S., Gardel M., Ma X., Adelstein R.S., Waterman C.M. (2009). Local cortical tension by myosin II guides 3D endothelial cell branching. Curr. Biol..

[58] Elliott H., Fischer R.S., Myers K.A., Desai R.A., Gao L., Chen C.S., Adelstein R.S., Waterman C.M., Danuser G. (2015). Myosin II controls cellular branching morphogenesis and migration in three dimensions by minimizing cell-surface curvature. Nat. Cell Biol..

[59] Parsons J.T., Horwitz A.R., Schwartz M.A. (2010). Cell adhesion: Integrating cytoskeletal dynamics and cellular tension. Nat. Rev. Mol. Cell Biol..

[60] Bryan B.A., Dennstedt E., Mitchell D.C., Walshe T.E., Noma K., Loureiro R., Saint-Geniez M., Campaigniac J.P., Liao J.K., D’Amore P.A. (2010). RhoA/ROCK signaling is essential for multiple aspects of VEGF-mediated angiogenesis. FASEB J..

[61] Huang L., Dai F., Tang L., Bao X., Liu Z., Huang C., Zhang T., Yao W. (2018). Distinct Roles For ROCK1 and ROCK2 in the Regulation of Oxldl-Mediated Endothelial Dysfunction. Cell Physiol. Biochem..

[62] Riento K., Ridley A.J. (2003). Rocks: Multifunctional kinases in cell behaviour. Nat. Rev. Mol. Cell Biol..

[63] Rikitake Y., Liao J.K. (2005). ROCKs as therapeutic targets in cardiovascular diseases. Expert Rev. Cardiovasc. Ther..

[64] Thumkeo D., Keel J., Ishizaki T., Hirose M., Nonomura K., Oshima H., Oshima M., Taketo M.M., Narumiya S. (2003). Targeted disruption of the mouse rho-associated kinase 2 gene results in intrauterine growth retardation and fetal death. Mol. Cell Biol..

[65] Shimizu Y., Thumkeo D., Keel J., Ishizaki T., Oshima H., Oshima M., Noda Y., Matsumura F., Taketo M.M., Narumiya S. (2005). ROCK-I regulates closure of the eyelids and ventral body wall by inducing assembly of actomyosin bundles. J. Cell Biol..

[66] Shimokawa H., Seto M., Katsumata N., Amano M., Kozai T., Yamawaki T., Kuwata K., Kandabashi T., Egashira K., Ikegaki I. (1999). Rho-kinase-mediated pathway induces enhanced myosin light chain phosphorylations in a swine model of coronary artery spasm. Cardiovasc. Res..

[67] Sato M., Tani E., Fujikawa H., Kaibuchi K. (2000). Involvement of Rho-kinase-mediated phosphorylation of myosin light chain in enhancement of cerebral vasospasm. Circ. Res..

[68] Mukai Y., Shimokawa H., Matoba T., Kandabashi T., Satoh S., Hiroki J., Kaibuchi K., Takeshita A. (2001). Involvement of Rho-kinase in hypertensive vascular disease: A novel therapeutic target in hypertension. FASEB J..

[69] Abe K., Shimokawa H., Morikawa K., Uwatoku T., Oi K., Matsumoto Y., Hattori T., Nakashima Y., Kaibuchi K., Sueishi K. (2004). Long-term treatment with a Rho-kinase inhibitor improves monocrotaline-induced fatal pulmonary hypertension in rats. Circ. Res..

[70] Satoh S., Utsunomiya T., Tsurui K., Kobayashi T., Ikegaki I., Sasaki Y., Asano T. (2001). Pharmacological profile of hydroxy fasudil as a selective rho kinase inhibitor on ischemic brain damage. Life Sci..

[71] Zhao J., Zhou D., Guo J., Ren Z., Zhou L., Wang S., Xu B., Wang R. (2006). Effect of fasudil hydrochloride, a protein kinase inhibitor, on cerebral vasospasm and delayed cerebral ischemic symptoms after aneurysmal subarachnoid hemorrhage. Neurol. Med. Chir..

[72] Liao J.K., Seto M., Noma K. (2007). Rho kinase (ROCK) inhibitors. J. Cardiovasc. Pharmacol..

[73] Barry D.M., Koo Y., Norden P.R., Wylie L.A., Xu K., Wichaidit C., Azizoglu D.B., Zheng Y., Cobb M.H., Davis G.E. (2016). Rasip1-Mediated Rho GTPase Signaling Regulates Blood Vessel Tubulogenesis via Nonmuscle Myosin II. Circ. Res..

[74] Sims D.E. (1986). The pericyte—A review. Tissue Cell.

[75] Peppiatt C.M., Howarth C., Mobbs P., Attwell D. (2006). Bidirectional control of CNS capillary diameter by pericytes. Nature.

[76] Hartmann D.A., Coelho-Santos V., Shih A.Y. (2022). Pericyte Control of Blood Flow Across Microvascular Zones in the Central Nervous System. Annu. Rev. Physiol..

[77] Wang Y., Zheng X.R., Riddick N., Bryden M., Baur W., Zhang X., Surks H.K. (2009). ROCK isoform regulation of myosin phosphatase and contractility in vascular smooth muscle cells. Circ. Res..

[78] Penzes M., Turos D., Mathe D., Szigeti K., Hegedus N., Rauscher A.A., Toth P., Ivic I., Padmanabhan P., Pal G. (2020). Direct myosin-2 inhibition enhances cerebral perfusion resulting in functional improvement after ischemic stroke. Theranostics.

[79] Tang L., Dai F., Liu Y., Yu X., Huang C., Wang Y., Yao W. (2018). RhoA/ROCK signaling regulates smooth muscle phenotypic modulation and vascular remodeling via the JNK pathway and vimentin cytoskeleton. Pharmacol. Res..

[80] Ashraf J.V., Al Haj Zen A. (2021). Role of Vascular Smooth Muscle Cell Phenotype Switching in Arteriogenesis. Int. J. Mol. Sci..

[81] Doe C., Bentley R., Behm D.J., Lafferty R., Stavenger R., Jung D., Bamford M., Panchal T., Grygielko E., Wright L.L. (2007). Novel Rho kinase inhibitors with anti-inflammatory and vasodilatory activities. J. Pharmacol. Exp. Ther..

[82] Yao L., Chandra S., Toque H.A., Bhatta A., Rojas M., Caldwell R.B., Caldwell R.W. (2013). Prevention of diabetes-induced arginase activation and vascular dysfunction by Rho kinase (ROCK) knockout. Cardiovasc. Res..

[83] Shimamura T., Zhu Y., Zhang S., Jin M.B., Ishizaki N., Urakami A., Totsuka E., Kishida A., Lee R., Subbotin V. (1999). Protective role of nitric oxide in ischemia and reperfusion injury of the liver. J. Am. Coll. Surg..

[84] Cohen P., Alessi D.R. (2013). Kinase drug discovery—What’s next in the field?. ACS Chem. Biol..

[85] Aref Z., de Vries M.R., Quax P.H.A. (2019). Variations in Surgical Procedures for Inducing Hind Limb Ischemia in Mice and the Impact of These Variations on Neovascularization Assessment. Int. J. Mol. Sci..

[86] Yang Y., Tang G., Yan J., Park B., Hoffman A., Tie G., Wang R., Messina L.M. (2008). Cellular and molecular mechanism regulating blood flow recovery in acute versus gradual femoral artery occlusion are distinct in the mouse. J. Vasc. Surg..

[87] Drewry D.H., Willson T.M., Zuercher W.J. (2014). Seeding collaborations to advance kinase science with the GSK Published Kinase Inhibitor Set (PKIS). Curr. Top Med. Chem..

[88] Zhang J.H., Chung T.D., Oldenburg K.R. (1999). A Simple Statistical Parameter for Use in Evaluation and Validation of High Throughput Screening Assays. J. Biomol. Screen..

[89] Pelz O., Gilsdorf M., Boutros M. (2010). web cellHTS2: A web-application for the analysis of high-throughput screening data. BMC Bioinform..

[90] Chen Y., Lu B., Yang Q., Fearns C., Yates J.R., Lee J.D. (2009). Combined integrin phosphoproteomic analyses and small interfering RNA-based functional screening identify key regulators for cancer cell adhesion and migration. Cancer Res..

[91] Kireeva M.L., Lam S.C., Lau L.F. (1998). Adhesion of human umbilical vein endothelial cells to the immediate-early gene product Cyr61 is mediated through integrin alphavbeta3. J. Biol. Chem..

[92] Abengozar M.A., de Frutos S., Ferreiro S., Soriano J., Perez-Martinez M., Olmeda D., Marenchino M., Canamero M., Ortega S., Megias D. (2012). Blocking ephrinB2 with highly specific antibodies inhibits angiogenesis, lymphangiogenesis, and tumor growth. Blood.

[93] Stock J., Kazmar T., Schlumm F., Hannezo E., Pauli A. (2022). A self-generated Toddler gradient guides mesodermal cell migration. Sci. Adv..

[94] Carpentier G., Berndt S., Ferratge S., Rasband W., Cuendet M., Uzan G., Albanese P. (2020). Angiogenesis Analyzer for ImageJ—A comparative morphometric analysis of “Endothelial Tube Formation Assay” and “Fibrin Bead Assay”. Sci. Rep..

[95] Couffinhal T., Silver M., Zheng L.P., Kearney M., Witzenbichler B., Isner J.M. (1998). Mouse model of angiogenesis. Am. J. Pathol..

[96] Rikitake Y., Kim H.H., Huang Z., Seto M., Yano K., Asano T., Moskowitz M.A., Liao J.K. (2005). Inhibition of Rho kinase (ROCK) leads to increased cerebral blood flow and stroke protection. Stroke.

[97] Neale J.P.H., Pearson J.T., Thomas K.N., Tsuchimochi H., Hosoda H., Kojima M., Sato T., Jones G.T., Denny A.P., Daniels L.J. (2020). Dysregulation of ghrelin in diabetes impairs the vascular reparative response to hindlimb ischemia in a mouse model; clinical relevance to peripheral artery disease. Sci. Rep..

[98] Martello A., Mellis D., Meloni M., Howarth A., Ebner D., Caporali A., Al Haj Zen A. (2018). Phenotypic miRNA Screen Identifies miR-26b to Promote the Growth and Survival of Endothelial Cells. Mol. Ther. Nucleic Acids.

[99] Leikert J.F., Rathel T.R., Muller C., Vollmar A.M., Dirsch V.M. (2001). Reliable in vitro measurement of nitric oxide released from endothelial cells using low concentrations of the fluorescent probe 4,5-diaminofluorescein. FEBS Lett..

[100] Gu L., Lian D., Zheng Y., Zhou W., Gu J., Liu X. (2020). Echinacoside-induced nitric oxide production in endothelial cells: Roles of androgen receptor and the PI3K-Akt pathway. Int. J. Mol. Med..

