# Supplementary material for: Selective ROCK Inhibitor Enhances Blood Flow Recovery after Hindlimb Ischemia
Source: Int J Mol Sci. 2023 Sep 22;24(19):14410. doi: 10.3390/ijms241914410 (PMC10572734; doi:10.3390/ijms241914410)
Supplement: Supplementary file 1 [file ijms-24-14410-s001.zip › ijms-2586524-supplementary.pdf]

Figure S1

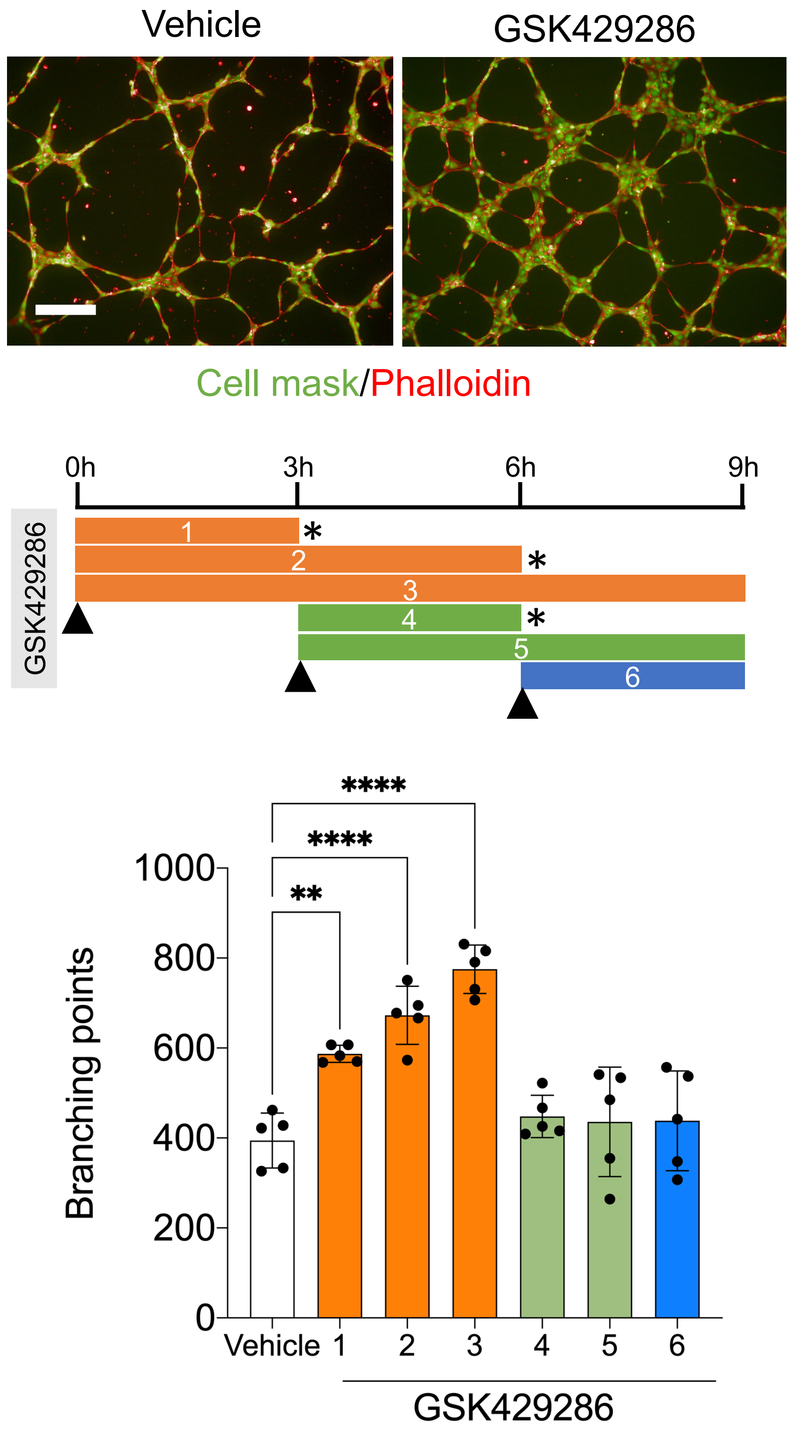

Figure S1. Effect of the ROCK inhibitor "GSK429286" incubated for different time periods during endothelial tube formation assay. HUVEC were seeded under vascular growth conditions (EMG2 media). GSK429286 (10 μM) was added at different time points: 0h, 3h or 6h after seeding (arrow) and incubated for 3h, 6 h, or 9h. The compound was washed-out by changing media at the time points: 3h or 6h (asterisk). All six conditions were fixed and stained after 9 hours of seeding. *Upper,* representative fluorescent images of endothelial tubes stained with phalloidin (actin, red) and cell mask (cell body, green). GSK429286 was incubated for the first three hours of assay (condition 1), Scale bar = 100 μm. Branching points were quantified using metamorph image analysis software. Data were expressed as mean ± SD; n = 5 per condition; one-way ANOVA followed by Bonferroni post hoc test, **p < 0.01 and ****p<0.0001 compared to vehicle.
